# Optimized deep learning ensemble using Fast Osprey algorithm for accurate lymphoblastic leukemia detection

**DOI:** 10.3389/fmed.2026.1812486

**Published:** 2026-05-01

**Authors:** Narinder Kaur, Shakir Khan, Bobbinpreet Kaur, Amal Alomran, Sultan Ahmad, Thamer Alshammari, Fahad Omar Alomary

**Affiliations:** 1 Department of Computer Science &; Engineering, Chandigarh University, Mohali, Punjab, India; 2 College of Computer and Information Sciences, Imam Mohammad Ibn Saud Islamic University (IMSIU), Riyadh, Saudi Arabia; 3 Department of Computer Science, College of Computer Engineering and Sciences, Prince Sattam Bin Abdulaziz University, Alkharj, Saudi Arabia; 4 School of Computer Science and Engineering, Lovely Professional University, Phagwara, Punjab, India; 5 Department of Computer Science, Saudi Electronic University, Riyadh, Saudi Arabia

**Keywords:** Acute Lymphoblastic Leukemia (ALL), computer-aided diagnosis (CAD), deep learning, ensemble learning, Fast Osprey Optimization (FOO), medical image analysis

## Abstract

**Introduction:**

Acute Lymphoblastic Leukemia (ALL) is a hematological malignancy, which is life-threatening and demands rapid and precise diagnosis to either enhance or worsen the survival chances. Traditional diagnostic methods, especially the manual microscopic examination, are labor-intensive and subject to inter-observer variability. Even though deep learning models have been shown to achieve good performance in automated detection, single-model structures tend to be prone to overfitting and under-generalize to heterogeneous datasets, with low interpretability. Hence, an effective and responsive computer-aided diagnostic (CAD) platform is required to promote the reliability of diagnostics.

**Methods:**

We introduce a new ensemble-based model that can be trained on a combination of several state-of-the-art convolutional neural networks (CNNs), such as EfficientNetB3, EfficientNetV2B3, and EfficientNetV2B1, and optimized with Fast Osprey Optimization (FOO), a bio-inspired algorithm that dynamically assigns optimal ensemble weights. An extensive dataset was formed through the combination of all publicly available datasets, and thereafter, data augmentation was used to address the issue of class imbalance and to improve the generalization of the model. The FOO algorithm is a model contribution optimization algorithm that is used in the training process to enhance predictive robustness and computational efficiency.

**Results:**

The proposed FOO-Ensemble model outperformed all baseline architectures. It achieved an accuracy of 97.76%, a precision of 98.13%, a recall of 97.71%, and an F1-score of 97.83%. In addition to improved classification performance, the ensemble approach reduced inference time compared to individual models. Comparative analysis with recent state-of-the-art methods further demonstrated the robustness, scalability, and superior generalization capability of the proposed framework.

**Conclusion:**

The results demonstrate the usefulness of using deep learning ensembles with bio-inspired optimization in trustworthy ALL detection. A dynamic weighting mechanism improves stability and minimizes the risks of overfitting of standalone models. The higher diagnostic quality and computational capability have high chances of real clinical application. The suggested FOO-Ensemble framework is a scalable and reliable CAD model that will be able to assist hematopathologists in making early and accurate diagnoses of ALL, which will ultimately result in the provision of better patient outcomes.

## 1 Introduction

**Figure 1:**
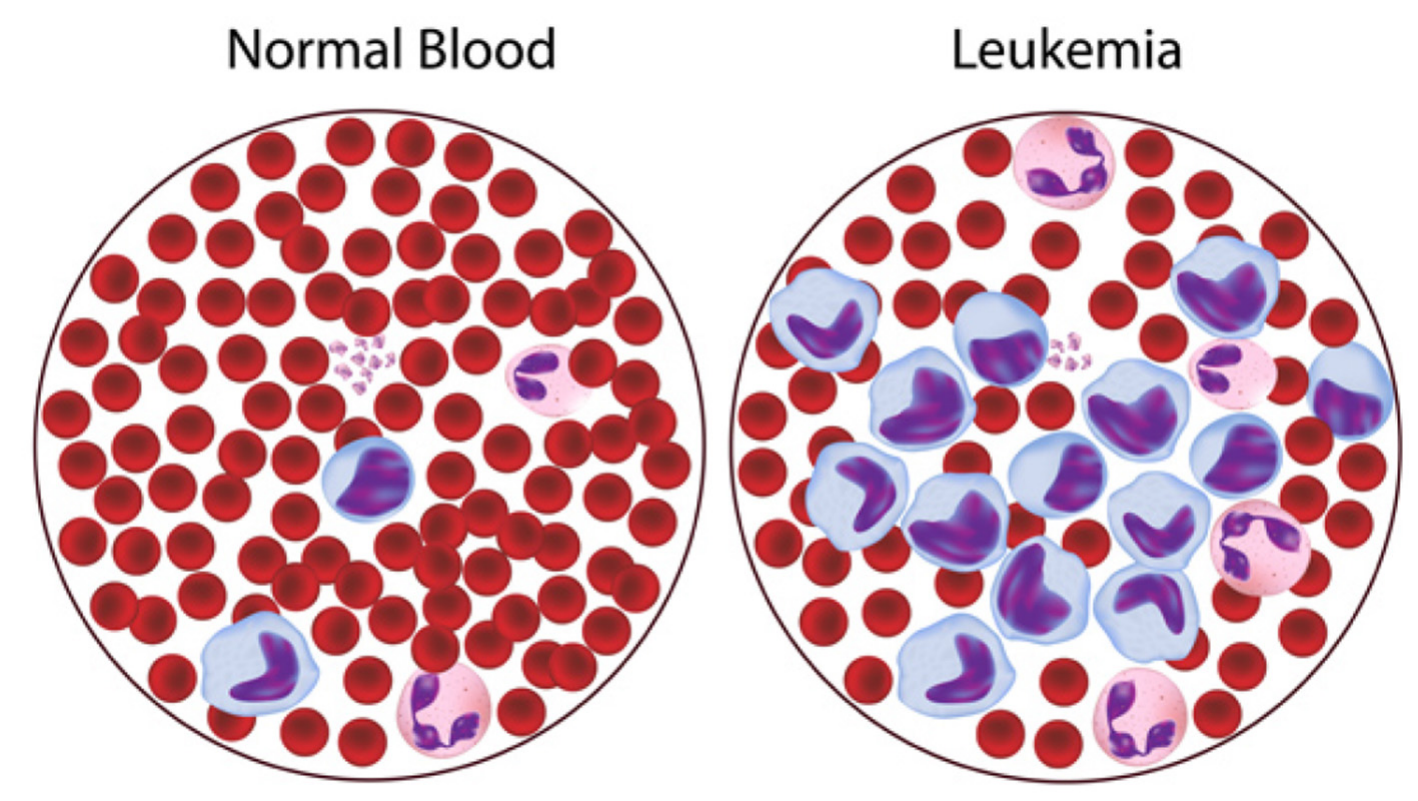
Comparison between normal blood and leukemia-affected blood (2).

Acute Lymphoblastic Leukemia (ALL), a highly aggressive hematological malignancy, poses significant challenges in early identification and diagnosis due to the morphological similarities between malignant and normal lymphoblast cells. ALL is a serious blood malignancy in which immature white blood cells called lymphoblasts grow too quickly in the bone marrow (1). These abnormal cells grow and take the place of good blood cells, which can lead to anemia, a higher risk of infection, and bleeding, as shown in Figure 1. The left panel shows normal blood, with a balanced proportion of red blood cells (erythrocytes) and a few white blood cells (leukocytes). The right panel illustrates leukemia, where abnormal and excessive immature white blood cells (blasts, shown in purple/blue) crowd out normal cells, disrupting healthy blood function. They can also quickly spread to organs like the liver, spleen, and central nervous system. ALL is the most common malignancy in children. However, it can also happen in adults, and the outlook is usually worse. Acute lymphocytic leukemia (ALL) is the most prevalent childhood cancer (0–14 years old). Young children have a greater probability of survival by over 50% in the past 50 years. The current survival rate stands at 65.3%; among children below 5 years, the survival rate stands at 90.4%. Despite progress in chemotherapy, radiation therapy, and stem cell transplantation, timely and accurate diagnosis is crucial for improving treatment outcomes and survival rates (2).

It is a rare cancer, making up less than 0.5% of all cancers in the United States (3). The average lifetime risk of developing ALL is approximately 1 in 1,000, with a slightly higher incidence in boys than in females and a greater frequency in White communities compared to Black communities. Even though ALL is rare, it is nonetheless clinically important because of its distinct risk profile that changes with age. In the past, doctors had to look at peripheral blood smears or bone marrow biopsy materials under a microscope to figure out if someone had ALL. Standard diagnostic methods, such as manual microscopic examination, are sometimes labor-intensive, prone to inter-observer variability, and can result in prolonged or inaccurate diagnosis (4). Even while this method works, it takes a lot of work, depends a lot on the pathologists' knowledge, and can be affected by differences between observers and fatigue. Recent developments in artificial intelligence, particularly deep learning, have shown great promise in automating and enhancing the accuracy of leukemia detection (5). Using convolutional neural networks (CNNs) and other deep learning frameworks makes it possible to directly extract discriminative features from microscopic blood smear images (6). Researchers have been looking into computer-aided diagnosis (CAD) systems that use artificial intelligence (AI) to help hematologists make more accurate diagnoses and ease their workload (7). Deep learning (DL), particularly CNNs, has emerged as a powerful tool for medical image analysis due to their ability to autonomously construct hierarchical features from raw image data, thereby obviating the necessity for manual feature engineering (8). This cuts down on the need for a lot of manual feature engineering. These models improve the accuracy and sensitivity of diagnoses and offer scalable and reliable solutions for clinical use. This helps hematologists make quick and effective decisions on how to care for patients. These models have demonstrated remarkable effectiveness in various medical imaging domains, including the detection of brain tumors, diabetic retinopathy, and hematological disorders such as ALL (9).

A lot of studies have used CNNs and transfer learning models like ResNet, DenseNet, Xception, and EfficientNet to tell the difference between ALL and other types of blood cells. They have gotten very high accuracy rates on benchmark datasets (10). Even so, these promising results do not mean that present methods do not still have big problems. Many models suffer from overfitting due to datasets that are small and unbalanced, which makes them less useful in real-world clinical settings when staining and imaging conditions change. Deep models can make real-time diagnostic applications harder to use because they are so complicated to compute (11, 12). Their “black-box” nature also makes it harder for doctors to understand what the forecasts mean. Additionally, individual CNN models may insufficiently capture the morphological diversity of leukemic cells, leading to misdiagnosis.

To address these limitations, ensemble learning has gained increased attention in the field of medical image analysis. By combining the best parts of different base learners, ensemble methods reduce volatility, improve resilience, and increase classification accuracy (13). When used with optimization techniques, ensemble frameworks can change the weights of models in real time to improve performance on a number of evaluation measures. This approach may effectively mitigate dataset variability, reduce the misclassification of morphologically similar cells, and improve the reliability of clinical decision support in the setting of ALL diagnosis.

This paper presents a precision-focused computer-aided detection (CAD) system for ALL detection, integrating deep neural networks with ensemble learning and enhancing ensemble weights through the Fast Osprey Optimization (FOO) algorithm. The proposed methodology aims to enhance diagnostic accuracy, improve generalization across datasets, and provide interpretable results for treatment decision-making. This study employs the discriminative power of deep CNNs and the robustness of ensemble optimization to enhance dependable AI-assisted hematological diagnosis for practical implementation. The proposed model is intended as a decision support system to assist clinicians in leukemia classification and not as a standalone diagnostic tool. Further clinical validation and risk assessment are required before real-world deployment.

### 1.1 Motivation

ALL is a life-threatening hematological malignancy necessitating prompt and precise diagnosis for appropriate treatment. Deep neural networks show promise for automating feature extraction and improving diagnostic accuracy, but individual models often have problems like overfitting and inconsistent performance. Ensemble learning is an appropriate mechanism to improve robustness and generalization by integrating the main features of many models. Using ensemble methods with deep neural networks makes them more accurate, helps them operate better in new situations, and gives reliable results in the clinic. Also, using advanced metaheuristic algorithms to optimize ensemble weights can greatly improve classification performance, making sure that it works well in real-life diagnostic circumstances. The goal of this work is to provide a very accurate, dependable, and easy-to-understand method for ALL detection that makes it easier to intervene early and reduces diagnostic uncertainty. This will improve patient outcomes and the efficiency of healthcare. This work seeks to create a precise detection framework for ALL using deep neural networks and optimized ensemble learning.

### 1.2 Major contributions

The main contributions of this study are summarized as follows:

1. Proposed a novel FOO-based ensemble optimization framework for Acute Lymphoblastic Leukemia (ALL) detection.
2. Developed a dynamic convex weight learning mechanism that adaptively combines multiple deep learning models by optimizing their contributions.
3. Introduced a non-linear rank-based fusion strategy that integrates exponential, hyperbolic tangent, and sigmoid transformations to enhance the robustness of prediction aggregation beyond conventional averaging or voting schemes.
4. Designed an efficient and computationally lightweight ensemble architecture that achieves faster convergence and reduced inference time compared to traditional metaheuristic-based ensemble methods.

## 2 Related work

Much research has been conducted in trying to establish the method of diagnosing lymphoblastic leukemia, which demonstrated limited generalizability and a considerable dependence on attributes delineated by specialists. Because deep learning is moving so quickly, especially CNNs and transfer learning models, researchers have been using automated frameworks that can directly learn hierarchical features from blood smear images more and more. These technologies have shown better accuracy, reliability, and speed (14). Every traditional machine learning classifier (Naive Bayes, Support Vector Machine, and K-Nearest Neighbor) was used in the work to classify images on the basis of selected features. The use of the Ant Colony Optimization (ACO) as a form of feature selection was introduced to improve the performance of the classification so that the most relevant set of features of the segmented cell regions could be selected. The experimental results revealed that the NB classifier outperformed the other models, achieving an accuracy of 96.15%, sensitivity of 97.56%, and specificity of 94.59%. This highlights the potential of combining optimization algorithms with traditional classifiers to improve diagnostic performance in hematological image analysis (15). The researchers have examined automated methods for differentiating the two using blood smear images. The ALL-IDB2 dataset, which comprises 110 training images of both blast and healthy cells, has been used to classify ALL. The BCCD dataset, which comprises 364 blood smear images (349 of which show different types of WBCs), has been used for WBC identification and categorization. Image segmentation was performed using a hue-saturation-value (HSV) color-based watershed approach, followed by deep learning techniques for classification. The VGG16 convolutional neural network (CNN) architecture has been utilized for the classification and enumeration of white blood cell (WBC) types from segmented images, followed by testing to identify WBC blasts. This automated system uses WBC detection, blast identification, and CNN-based classification to make it easier to tell the difference between leukemia and leukemoid reactions. The overall accuracy obtained is 96.84%. An intelligent deep learning-based model design was presented (16). The model showed a considerable improvement in performance, justified by the values of metrics obtained. Unlike traditional image processing techniques that rely on handcrafted features, the deep learning approach demonstrated superior performance in blast cell detection, achieving a classification accuracy of 95% with a custom ALL-NET model. The framework's ability to operate directly on the entire dataset, without requiring feature vector selection, contributed to its efficiency and reduced error rates. This approach not only supports faster screening but also shows potential as a theoretical foundation for computer-aided diagnosis tools in ALL detection. A similar approach presented in Das and Meher (17) implemented a novel probability-based weight factor to improve CNN performance. A comparative analysis has been presented by varying the train vs. test split and keeping ratios as 70:30, 60:40, and 50:50. The proposed model achieved 97.18% accuracy on the ALL-IDB2 dataset when evaluated with a 70% training and 30% testing split. Under a 50% training and 50% testing configuration, the model still demonstrated strong generalization, attaining 96.00% accuracy on ALL-IDB2, respectively. These outcomes highlight the robustness of CNN architectures in achieving state-of-the-art performance across different training-testing splits for ALL detection tasks (16). A custom CNN-based model called ALLNET, using open-source datasets and run on Google Colaboratory with Nvidia Tesla P-100 GPU support, was trained. The model achieved an accuracy of 95.54%, a specificity of 95.81%, a sensitivity of 95.91%, a precision of 96%, and an F1-score of 95.43%. The CNN-based algorithm is also able to detect the blast cells in a short time and with high accuracy, unlike other traditional image processing techniques that use manual feature engineering. The authors (18) presented a novel approach using the You Only Look Once (YOLOv4) algorithm, which aimed at classifying the data belonging to 2 classes. The precision value obtained with the proposed approach is 95.57%. Along with precision considerations, the proposed method considered the time taken and the reduction of errors for the process (18). An ensemble learning system based on ResNet101-9 was developed for the classification of Acute Lymphoblastic Leukemia (ALL) in microscopic images. The model included nine ResNet-101 networks that had been trained separately by utilizing a majority voting method. The Taguchi experimental method was used to find the best hyperparameters. We used the C-NMC dataset to train and test. The experimental results showed that the ResNet101-9 ensemble had an accuracy of 85.11% and an F1-score of 88.94, which was better than each individual ResNet-101 model in all important areas, such as precision, recall, and specificity. This highlights the potential of ensemble deep learning frameworks to enhance diagnostic precision in leukemia classification. A MobileNetV2-based system (19) achieved 97% accuracy, demonstrating high sensitivity and specificity in identifying multiple ALL subtypes. Beyond classification, the study introduced telediagnosis software that enables real-time clinical support, thereby facilitating timely and accurate leukemia diagnosis. The approach emphasizes accessibility and affordability, addressing the global burden of ALL. However, significant challenges persist due to the high variability in blast cell patterns, shapes, and textures, which complicates robust detection. To overcome these limitations, multi-DNN models and tele-diagnostic applications have been designed for efficient diagnostic assistance. While existing methods show promise, a comprehensive and generalized framework for image analysis, region-of-interest detection, and blast cell quantification remains an open research direction. A novel ensemble framework (20), RanBALL, has been proposed for cost-effective and accurate B-ALL subtype identification. The model leverages random projection (RP) for dimensionality reduction while preserving patient-to-patient distance relationships, combined with ensemble learning for robust classification. Evaluated on a cohort of over 1,700 B-ALL patients, RanBALL achieved strong performance with 0.93 accuracy, a 0.93 F1-score, and a 0.93 Matthews correlation coefficient (MCC), outperforming state-of-the-art methods such as ALLSorts across all metrics. This highlights its potential as an efficient diagnostic tool for B-ALL subtyping. The study proposed by Himel et al. used a publicly accessible microscopic blood smear image dataset (ALL-IDB or equivalent leukemia classification benchmarks) (21). The approach uses transfer learning to fuse deep features from separate networks to generate a robust feature representation from numerous pre-trained deep learning models. Machine learning classifiers like SVMs classify this fused feature vector. Using complementary model properties, the ensemble technique increases diagnostic performance. Automatic leukemia detection was possible with the suggested method's 98–99% classification accuracy.

Recent initiatives have investigated sophisticated CAD systems to tackle diagnostic difficulties. A Deep Dilated Residual Convolutional Neural Network (DDRNet) (22) was developed for the classification of blood cell images into eosinophils, lymphocytes, monocytes, and neutrophils. The model uses Deep Residual Dilated Blocks (DRDB), Global and Local Feature Enhancement Blocks (GLFEB), and Channel and Spatial Attention Blocks (CSAB) to overcome vanishing gradients, provide more feature discrimination, and balance the input of shallow and deep features. After testing an image dataset of 16,249 images on Kaggle, the DDRNet reached an accuracy of 91.98 cred and an F1-score of 0.96, which outperforms traditional methods with low computation cost. An ensemble-ALL model (23) was created by integrating leading CNN architectures, such as InceptionV3, EfficientNetB4, ResNet50, CONV_POOL-CNN, ALL-CNN, Network in Network, and AlexNet. The top four models were connected with squeeze-and-excitation (SE) modules, and the two most effective SE-embedded models were subsequently blended via Bayesian optimization. The ensemble-ALL model, assessed using a publicly accessible dataset, attained an accuracy, precision, recall, and F1-score of 96.26%, in addition to a kappa score of 91.36%, exceeding state-of-the-art methodologies. This illustrates its capability as a dependable CAD tool to aid medical professionals in the prompt diagnosis and treatment of ALL. A two-stage CAD architecture (24) was presented for the detection of ALL, integrating feature extraction and classification. Features were retrieved utilizing three transfer learning models—InceptionResNetV2, DenseNet121, and VGG16—and consolidated through a Global Average Pooling layer before classification with a linear SVM. The experimental evaluation revealed robust performance, with a maximum accuracy of 91.63% attained via feature fusion from all three models. An extensively optimized CNN (25) was developed for the early identification of Acute Lymphoblastic Leukemia (ALL), comprising five convolutional blocks with thirteen convolutional layers and five max-pooling layers. The model was optimized over 30 epochs, using a batch size of 32 and two optimizers: Adam and Adamax. The results indicated that Adam outperformed Adamax, attaining an accuracy of 0.96 and a precision of 0.95, whereas Adamax achieved an accuracy of 0.91. Table 1 summarizes various existing methods.

**Table 1:** Summary of existing methods for all detection with key contributions and constraints.

Study	Method	Contribution	Constraint
Das et al. (17)	CNN + probabilistic weighting	Introduced a weighted CNN framework for improved classification performance	Limited generalization due to a small dataset
Khandekar et al. (18)	YOLOv4	Applied object detection for leukemia cell classification	Sensitive to bounding box quality affecting detection accuracy
Chen et al. (28)	ResNet101 ensemble	Demonstrated the effectiveness of ensemble deep learning for classification	High computational cost with moderate accuracy
Sriram et al. (15)	HSV + VGG16 CNN	Combined color-based segmentation with CNN classification	Dependency on color space; limited robustness
Sampathila et al. (16)	ALL-NET (custom CNN)	Developed task-specific CNN architecture for ALL detection	Lack of external validation and generalization
Khan et al. (19)	MobileNetV2 + telediagnosis	Proposed lightweight model for real-time clinical support	May miss complex feature representations
Huang et al. (23)	Ensemble-ALL (SE + Bayesian optimization)	Integrated multiple CNNs with optimization for improved performance	Increased training complexity and computational overhead
Hasanath et al. (24)	Two-stage CAD (multi-CNN + SVM)	Combined deep feature extraction with machine learning classification	Complex pipeline; difficult deployment
Anand et al. (25)	Deep CNN (13 convolutional layers)	Designed a deeper CNN for enhanced feature extraction	Risk of overfitting without optimization
El et al. (14)	ML classifiers + ACO feature selection	Improved feature selection using optimization techniques	Limited feature representation capability of traditional ML methods

Despite the substantial progress achieved by deep learning approaches in the detection of ALL, major research limitations remain. Initial research employing manually constructed features and rudimentary classifiers demonstrated shortcomings in robustness and generalizability. In contrast, modern CNN-based and transfer learning methodologies, despite their elevated accuracy, often face issues such as overfitting, limited interpretability, and dependence on particular datasets. Additionally, several contemporary research studies employ single-model architectures or ensemble approaches with fixed weights, thus constraining their adaptability across diverse clinical datasets. The absence of systematic weight optimization, limited cross-dataset validation, and challenges in mitigating class imbalance undermine the therapeutic significance of current models. These shortcomings highlight the need for advanced ensemble-based frameworks that employ optimization-driven weight distribution, enhanced interpretability, and greater generalization to practical diagnostic settings.

## 3 Proposed research framework

This research delineates a four-phase methodology for the automated categorization and detection of ALL disease by the utilization of microscopic blood smear images. The suggested approach, as shown in Figure 3, systematically includes preparing the dataset, balancing and adding to the classes, training a deep learning-based base learner, and using Fast Osprey Optimization (FOO) to find the best rank-optimized ensemble strategy. The ultimate goal is to get very accurate classifications of four clinically important groups: Benign, Early Pre-B, Pre-B, and Pro-B.

### 3.1 Dataset and its acquisition

**Figure 2:**
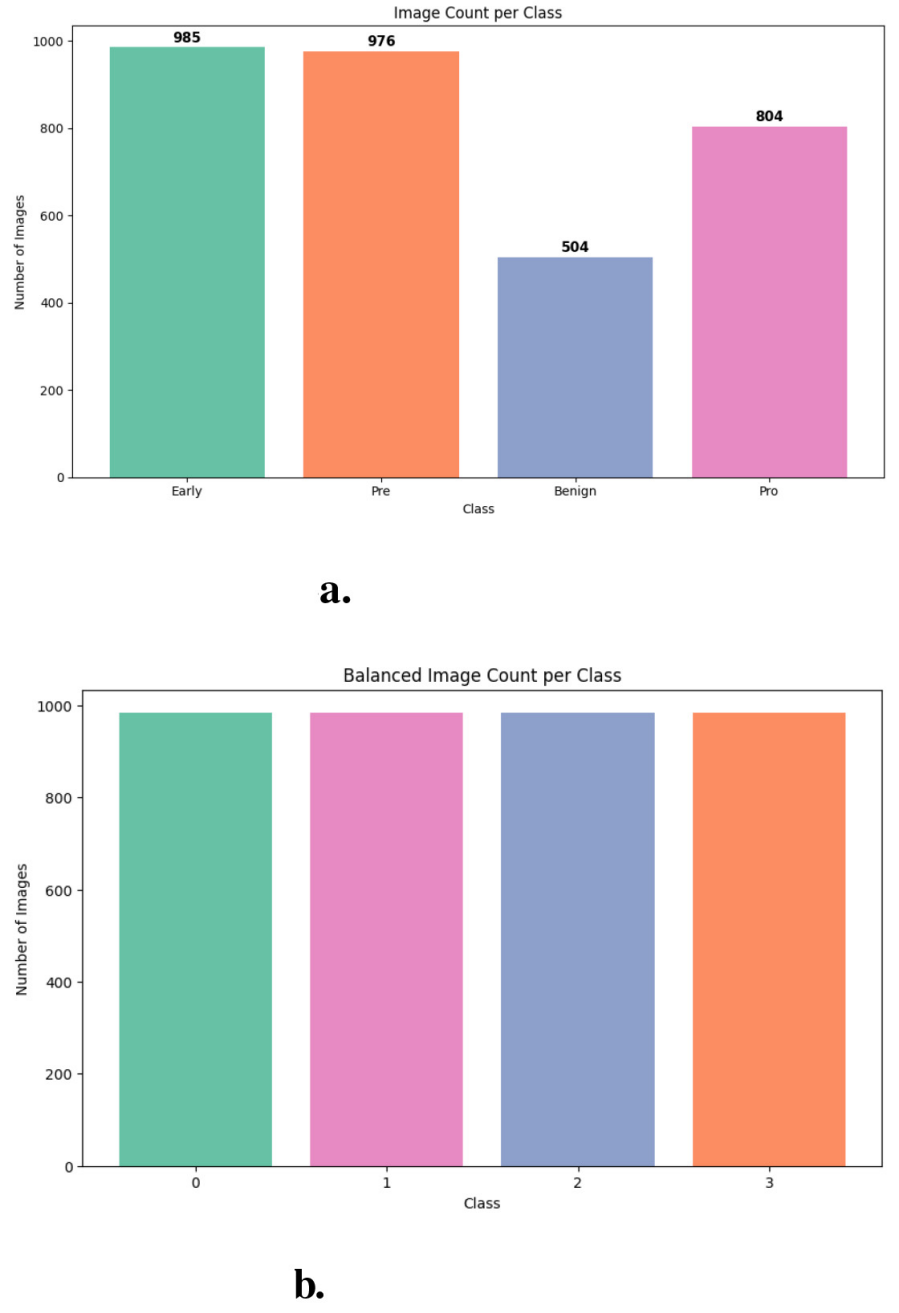
**(a)** Unbalanced dataset featuring four diagnostic catagories. **(b)** Balanced distribution of dataset.

The dataset named “Mehrad Aria Kaggle ALL dataset” comprises 3,256 peripheral blood smear PBS images collected from 89 individuals suspected of ALL. Laboratory-prepared and stained by skilled technicians, these images represent a valuable resource for deep learning-based diagnostics. The automated detection system is trained using the publicly available ALL image dataset by Mehrad Aria on Kaggle (26). Detailed description of the dataset is described in Table 2 and Figure 2. Leukemia can arise at different stages of blockage. Pro-B ALL is the earliest immature stage, Early Pre-B ALL is slightly more advanced, and Pre-B ALL is closer to becoming a normal B-cell. All images were prepared and stained by trained laboratory personnel following standard protocols. The dataset was ethically sourced, and all patient identifiers were removed to maintain confidentiality. Use of this dataset for research purposes complies with institutional and ethical guidelines, ensuring that the study adheres to privacy and data protection standards.

**Table 2:** Dataset overview.

Attribute	Description
Source	Kaggle—Acute Lymphoblastic Leukemia (ALL) image dataset (26)
Domain	Microscopic peripheral blood smear images.
Total images	3,256.
Patients	89 (25 healthy/benign diagnosis (hematogone); 64 with definitive ALL diagnosis).
Classes	1. Benign (504 images) 2. Early Pre-B ALL (985 images) 3. Pre-B ALL (963 images) 4. Pro-B ALL (804 images).

### 3.2 Data pre-processing and balancing

A structured data preprocessing pipeline is employed to ensure effective model training and evaluation. The dataset undergoes normalization, resizing, and labeling based on class-specific folders. To address class imbalance and improve generalization, data augmentation techniques such as rotation, translation, zooming, shearing, and flipping are applied. These transformations increase dataset diversity and reduce overfitting. The dataset is subsequently balanced to ensure uniform class distribution.

A multi-stage data preparation pipeline was utilized to ensure effective model training and reliable evaluation. The original dataset, comprising images classified into class-specific folders, underwent data augmentation. Various random transformations, such as rotations (up to 40°), horizontal and vertical shifts (20%), shear distortions, zooming, and horizontal flips, were applied. These changes artificially augmented the dataset size while providing controlled variability, thus mitigating overfitting and improving model generalization. The augmentation procedure was limited to producing more samples until each class attained a target of roughly 800 images, thus assuring class equilibrium. All augmented images were reallocated to their corresponding class folders, ensuring a consistent data structure. Each image was designated a categorized label that corresponds to its source directory. The resultant compilation of image-label pairs was organized and subsequently randomized to remove any ordering bias. This generated a randomized dataset that incorporated both original and modified samples. The dataset was divided for model evaluation using a stratified six-fold cross-validation approach. A new column was incorporated into the DataFrame to record fold assignments, initially set to -1. The dataset was divided into six mutually exclusive subsets using StratifiedKFold, maintaining the class distribution in each fold. Each image-label pair was allocated to one of the six folds, facilitating systematic rotation of training and validation sets across many studies. This method offered a thorough evaluation of model efficacy and reduced variation linked to a singular train-test split. Potential data leakage arising from augmentation prior to cross-validation may affect the absolute performance values; however, the relative comparison between models remains informative and indicative of their comparative effectiveness.

### 3.3 Base models

In the proposed framework, three variants of the EfficientNet family, namely EfficientNetB3, EfficientNetV2B3, and EfficientNetV2B1, are employed as base learners due to their superior efficiency in capturing multi-scale features from microscopic blood images with relatively fewer parameters (27). Three convolutional neural network architectures—EfficientNetB3, EfficientNetV2B3, and EfficientNetV2B1—are utilized as base learners due to their efficiency in capturing multi-scale features from microscopic images. Each model is initialized with pre-trained weights and fine-tuned for the four-class classification task. The final classification head is adapted using a Global Average Pooling layer, followed by fully connected layers and softmax activation.

Each network is initialized with pre-trained ImageNet weights to exploit transfer learning and subsequently fine-tuned on the Acute Lymphoblastic Leukemia (ALL) dataset. The input images are subjected to preprocessing like resizing, after which they are fed into the backbone networks. To adapt the models to the specific four-class classification problem, the final fully connected layer is replaced with a task-specific classifier comprising a *Global Average Pooling layer*, followed by a *dense layer of 256 units with ReLU activation*, a *dropout layer with rate 0.4* to reduce overfitting, and a final *softmax layer* with four neurons corresponding to the target classes {Benign, Early Pre-B, Pre-B, Pro-B}. Mathematically, given an input image $x_{i}\in {\mathbb{R}}^{128\times 128\times 3}$, the base learner *f*_θ_ with parameters θ produces a probability distribution over the classes given by Equation 1 (1),


$$\begin{array}{l} {\text{p}}_{i}=f_{\theta }\left(x_{i}\right)=\text{Softmax}\left(W\cdot h_{i}+b\right), \end{array}$$ (1)

where *h*_*i*_ represents the learned feature vector from the backbone, *W* and *b* are the trainable weights and bias of the classification head, and **p**_*i*_ = [*p*_*i*1_, *p*_*i*2_, *p*_*i*3_, *p*_*i*4_] denotes the predicted class probabilities such that $\overset{4}{\underset{j=1}{\sum }}p_{ij}=1$. The models are trained by minimizing the categorical cross-entropy loss represented by Equation 2:


$$\begin{array}{l} L=-\frac{1}{N}\overset{N}{\underset{i=1}{\sum }}\overset{4}{\underset{j=1}{\sum }}y_{ij}\log\left(p_{ij}\right), \end{array}$$ (2)

where *N* is the number of training samples, *y*_*ij*_ is the ground truth indicator (1 if the sample *i* belongs to class *j*, else 0), and *p*_*ij*_ is the predicted probability for class *j*.

### 3.4 Proposed Fast Osprey Optimization (FOO)

The proposed FOO algorithm is formulated as a task-specific ensemble weight optimization approach, leveraging convex combination principles to effectively integrate predictions from multiple models, rather than as a general-purpose metaheuristic optimizer. The proposed *Fast Osprey Optimization (FOO)* algorithm simplifies the traditional Osprey Optimization Algorithm (OOA) by adopting a single-phase, attractor-based update rule as elaborated in Algorithm 1. Each candidate solution (individual) is represented by a weight vector (Equation 3).


$$\begin{array}{l} w_{i}=[\alpha ,\beta ,\gamma ],\text{ where }w_{i}\in \Delta^{2}=\{w\in {\mathbb{R}}^{3}|\alpha +\beta +\gamma \}\\ \;=\{1,\;\alpha ,\beta ,\gamma \geq 0\}. \end{array}$$ (3)

Algorithm 1 Fast Osprey Optimization (FOO) for ensemble weighting. 
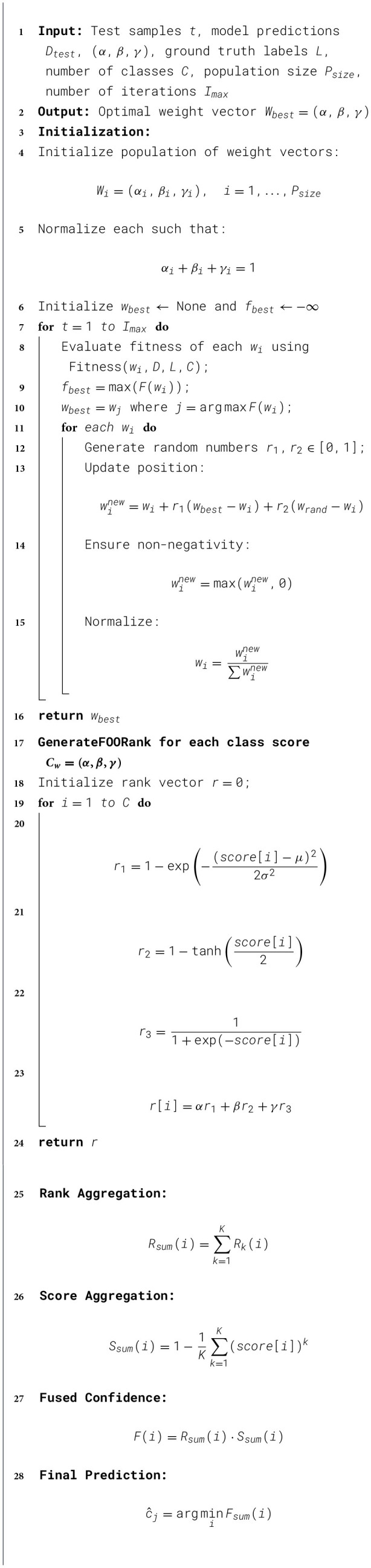

T at each iteration, the *ik*-th individual is updated by a convex combination of its current position, the global best position w^*^, and the population mean ${\bar{w}}^{t}$ given by Equation 4:


$$\begin{array}{l} w_{i}^{t+1}=\left(1-r_{1}-r_{2}\right)w_{i}^{t}+r_{1}w^{*}+r_{2}{\bar{w}}^{t} \end{array}$$ (4)

where $r_{1},r_{2}\sim U\left(0,1\right)$ are randomly sampled scalars from a uniform distribution. This formulation ensures that each update step is a stochastic contraction toward the best solution and the swarm's average behavior, promoting both convergence and mild exploration.

After each update, the solution is projected back onto the feasible region (the 3D probability simplex Δ^2^) as given in Equation 5:


$$\begin{array}{l} w_{i}\leftarrow \max\left(w_{i},0\right)w_{i}\leftarrow \frac{w_{i}}{\sum_{j}w_{ij}+\varepsilon } \end{array}$$ (5)

where ε is a small constant to avoid division by zero. This ensures that the updated weights remain valid for convex combination in ensemble model fusion.

Unlike traditional OOA, which models complex bio-inspired behavior such as dual exploration-exploitation phases, chaotic perturbations, and non-linear dynamics, FOO removes all such mechanisms. Instead, it emphasizes rapid convergence and computational efficiency, making it especially suitable for small-scale, constrained optimization tasks such as learning optimal fusion weights in ensemble classification models.

The Fast Osprey Optimization (FOO) algorithm is termed “fast” due to its computational simplicity, low-dimensional search space, and task-specific design. Unlike traditional Osprey Optimization Algorithm (OOA) and its variants, FOO avoids complex dual-phase exploration-exploitation mechanisms, chaotic maps, Lévy flights, and adaptive control parameters, all of which increase computational overhead. Instead, each individual is updated using a single stochastic convex combination of the global best solution and the population mean, followed by a simple projection onto the feasible simplex. Furthermore, FOO operates in a three-dimensional constrained space corresponding to the ensemble fusion weights (α, β, γ), which allows rapid convergence with a small population and few iterations. The combination of a minimal update rule, low-dimensionality, and absence of computationally expensive mechanisms makes FOO particularly fast and efficient for optimizing convex weight vectors in ensemble model fusion tasks.

### 3.5 Methodology

**Figure 3:**
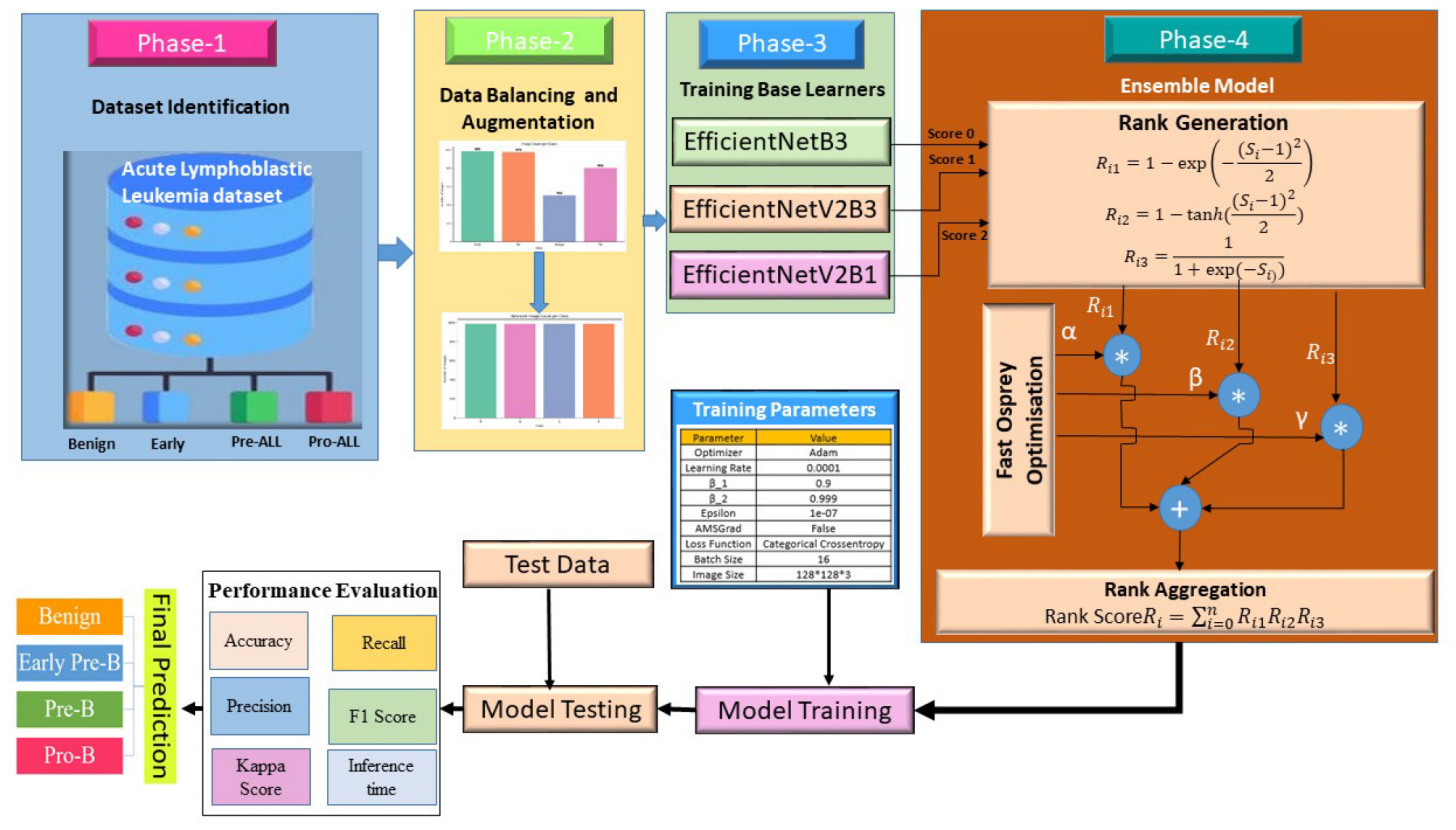
Proposed FOO-based ensemble model.

A detailed step-by-step methodology is elaborated in Figure 3 and Algorithm 1.

The framework begins with dataset identification, where the Acute Lymphoblastic Leukemia dataset is collected and categorized into four classes: Benign, Early Pre-B ALL, Pre-B ALL, and Pro-B ALL. This phase ensures that the raw image data is well-defined and systematically divided into relevant diagnostic categories. Such categorization is critical for enabling the deep learning model to differentiate between benign cases and progressive stages of leukemia. To overcome the challenges of class imbalance, which often biases model predictions toward majority classes, data balancing techniques are applied. This is followed by extensive data augmentation, which enhances the diversity of training samples by applying transformations such as rotation, flipping, shifting, and zooming. This step not only ensures that all classes contribute equally to the training process but also improves the model's generalization ability by preventing overfitting to a limited dataset. In the third phase, three state-of-the-art convolutional neural networks (CNNs) are employed as base learners: EfficientNetB3, EfficientNetV2B3, and EfficientNetV2B1. These architectures are chosen due to their efficiency in balancing accuracy and computational complexity. The models are trained using the Adam optimizer with fine-tuned hyperparameters, including a learning rate of 0.0001, β1 = 0.99, β2 = 0.999, epsilon = 1e-07, and a batch size of 16. The input images are resized to 128 × 128 × 3 dimensions, and categorical cross-entropy is used as the loss function. Each base model outputs a score for classification, which serves as the input for the subsequent ensemble phase. To enhance classification robustness, an ensemble strategy is employed, integrating the predictions of the three base learners.

This ensemble approach ensures that the model leverages the strengths of each base learner while mitigating individual weaknesses. The training process incorporates the above phases to build a robust ensemble model. Once training is completed, the test data is used for independent evaluation. Model testing outputs the predicted class labels corresponding to Benign, Early Pre-B ALL, Pre-B ALL, or Pro-B ALL.

To validate the model's performance, several evaluation metrics are employed, including accuracy, precision, recall, F1-score, Cohen's kappa score, and inference time. These metrics provide a comprehensive analysis of both predictive performance and computational efficiency. The integration of multiple performance indicators ensures the reliability of the model in practical clinical settings. The ensemble framework ultimately delivers the final prediction, classifying each input sample into one of the four diagnostic categories. By combining advanced CNN architectures, optimized ensemble learning, and rigorous evaluation, the proposed methodology aims to achieve high accuracy, robustness, and reliability in early ALL detection.

#### 3.5.1 Design of ensemble model

In Phase-4, the predictions from multiple base learners are aggregated into a single robust decision using a Fast Osprey Optimization (FOO)-based rank generation and fusion mechanism. Ensemble learning has emerged as a highly successful approach to mitigate the limitations of individual classifiers by integrating multiple models to achieve superior performance. A good ensemble needs advanced fusion mechanisms that can dynamically evaluate and combine predictions from different models. The proposed technique introduces a Fast Osprey Optimization (FOO)-based rank fusion strategy aimed at improving ensemble performance through the integration of non-linear rank transformations with a bio-inspired optimization framework. An ensemble in machine learning combines a lot of simple learners {*M*_1_, *M*_2_, …, *M*_*K*_} to make a meta-learner that works better. Ensemble methods can lower variance by averaging predictions and bias when basic models include different types of information, according to the bias-variance decomposition.

The sum rule, the product rule, and majority voting are all examples of traditional fusion rules that assume that all classifiers are equally important. Our methodology goes beyond simple probability averaging by using FOO-based non-linear rank transformations and weight optimization. Given a prediction score vector *S* = [*s*_1_, *s*_2_, …, *s*_*C*_], where *s*_*i*_ denotes the probability of class *i* and *C* is the total number of classes; the system transforms raw scores into **robust rank indicators**.

The transformation functions are inspired by probability theory and non-linear mappings:

1. **Exponential Decay Function (Gaussian-like smoothing)**

Captures the relative deviation from perfect confidence (*s*_*i*_ = 1) using Equation 6:


$$\begin{array}{l} R_{1}\left(i\right)=1-\exp\left(-\frac{{\left(score\left[i\right]-1\right)}^{2}}{2}\right) \end{array}$$ (6)

This emphasizes penalties for lower confidence values while preserving high-confidence predictions.

2. **Hyperbolic Tangent Function (bounded suppression)**

Provides smooth suppression of deviations while avoiding extreme fluctuations given through Equation 7:


$$\begin{array}{l} R_{2}\left(i\right)=1-\tanh\left(\frac{{\left(score\left[i\right]-1\right)}^{2}}{2}\right) \end{array}$$ (7)

This introduces non-linearity and stabilizes ranking under noisy conditions.

3. **Sigmoid Function (probabilistic activation)**

Models the natural probability saturation effect as given by Equation 8:


$$\begin{array}{l} R_{3}\left(i\right)=\frac{1}{1+\exp\left(-score\left[i\right]\right)} \end{array}$$ (8)

It emphasizes the asymptotic confidence of predictions.

Finally, the FOO-rank score for each class is derived as a convex combination (Equation 9):


$$\begin{array}{l} R\left(i\right)=\alpha R_{1}\left(i\right)+\beta R_{2}\left(i\right)+\gamma R_{3}\left(i\right),\;\alpha +\beta +\gamma =1 \end{array}$$ (9)

This makes sure that the fusion includes a number of different confidence levels, which lowers model bias and makes better use of diversity. Bio-inspired optimization algorithms simulate natural processes to solve high-dimensional search problems. The proposed method adapts the foraging strategy of ospreys, which dive rapidly toward prey while adjusting trajectories to balance *exploration* (searching new solutions) and *exploitation* (refining existing solutions).

The FOO algorithm mimics the diving and striking behavior of ospreys to balance exploration and exploitation during optimization. The FOO algorithm optimizes the weights (α, β, γ) as follows:

1. **Population Initialization**

A set of candidate solutions is randomly initialized as Equation 10:


$$w_{i}=(\alpha_{i},\beta_{i},\gamma_{i}),\;i=1,2,\ldots ,P_{\text{size}}$$ (10)

where *P* is the population size, and the weights are normalized such that (Equation 11):


$$\begin{array}{l} \alpha_{i}+\beta_{i}+\gamma_{i}=1,\;\alpha_{i},\beta_{i},\gamma_{i}\geq 0 \end{array}$$ (11)

2. **Exploration–Exploitation Balance**

Each candidate updates its position toward both the *best solution* and the *mean population* (Equation 12):


$$w_{i}^{\;new}=w_{i}+r_{1}(w_{\text{best}}-w_{i})+r_{2}(\bar{w}-w_{i})$$ (12)

where *r*_1_, *r*_2_~*U*(0, 1) are the random numbers, *W*_best_ is the current best candidate, and $\bar{W}$ is the mean of the population and is given by Equation 13


$$\begin{array}{l} \bar{w}=\frac{1}{P_{\text{size}}}\overset{P_{\text{size}}}{\underset{i=1}{\sum }}w_{i} \end{array}$$ (13)

3. **Convergence Criterion**

The process iterates until the fitness values converge, yielding the *optimal fusion weights*:


$$\left(\alpha^{*},\beta^{*},\gamma^{*}\right)=\arg\underset{\left(\alpha ,\beta ,\gamma \right)}{\max}\text{Accuracy}$$

This mimics the osprey's adaptive dive mechanics, where the trajectory adjustments improve strike precision.

For multiple models {*M*_1_, *M*_2_, …, *M*_*K*_}, the rank and score aggregation (Equations 14, 15 for input) *x*_*j*_ are defined as:


$$\begin{array}{l} R_{\text{sum}}\left(i\right)=\overset{K}{\underset{k=1}{\sum }}R_{\left(k\right)\left(i\right)}, \end{array}$$ (14)

$$\begin{array}{l} S_{\text{sum}}\left(i\right)=1-\frac{1}{K}\overset{K}{\underset{k=1}{\sum }}{\left(score\left[i\right]\right)}^{k}. \end{array}$$ (15)

The fused confidence is given by Equation 16:


$$\begin{array}{l} F\left(i\right)=R_{\text{sum}}\left(i\right)\cdot S_{\text{sum}}\left(i\right). \end{array}$$ (16)

The predicted class is given by Equation 17:


$$\begin{array}{l} {ĉ}_{j}=\arg\underset{i}{\min}R_{\text{sum}}\left(i\right). \end{array}$$ (17)

Finally, the ensemble classification accuracy across *N* test samples is defined as Equation 18:


$$\begin{array}{l} \text{Accuracy}=\frac{1}{N}\overset{N}{\underset{j=1}{\sum }}I\left({ĉ}_{j}=y_{j}\right), \end{array}$$ (18)

where *y*_*j*_ denotes the true class label, and *I*(·) is the indicator function (Equation 19):


$$I({\hat{c}}_{j}=y_{j})=\{\begin{array}{ll} 1, & \text{if }{\hat{c}}_{j}=y_{j},\\ 0, & \text{otherwise}. \end{array}$$ (19)

## 4 Experimental Results

### 4.1 Experimental Setup

All experiments were conducted using Python with TensorFlow/Keras deep learning libraries. The models were trained using the Adam optimizer with the hyperparameters described in the manuscript. The experiments were executed on a system equipped with GPU acceleration using NVIDIA Tesla P100 on Google Colaboratory, which significantly facilitated deep learning model training and evaluation. Standard libraries, including NumPy, Pandas, Scikit-learn, and Matplotlib, were used for data preprocessing, evaluation, and visualization.

### 4.2 Training parameters

Table 3 presents the training parameters employed to optimize the deep learning model for acute lymphoblastic leukemia classification.

**Table 3:** Training parameters used for optimizing the proposed method.

Parameter	Value
Optimizer	Adam
Learning rate	0.0001
β_1_	0.9
β_2_	0.999
Epsilon	1e-07
AMSGrad	False
Loss function	Categorical crossentropy
Batch size	16
Image size	128*128*3

### 4.3 Quantitative results

**Figure 4:**
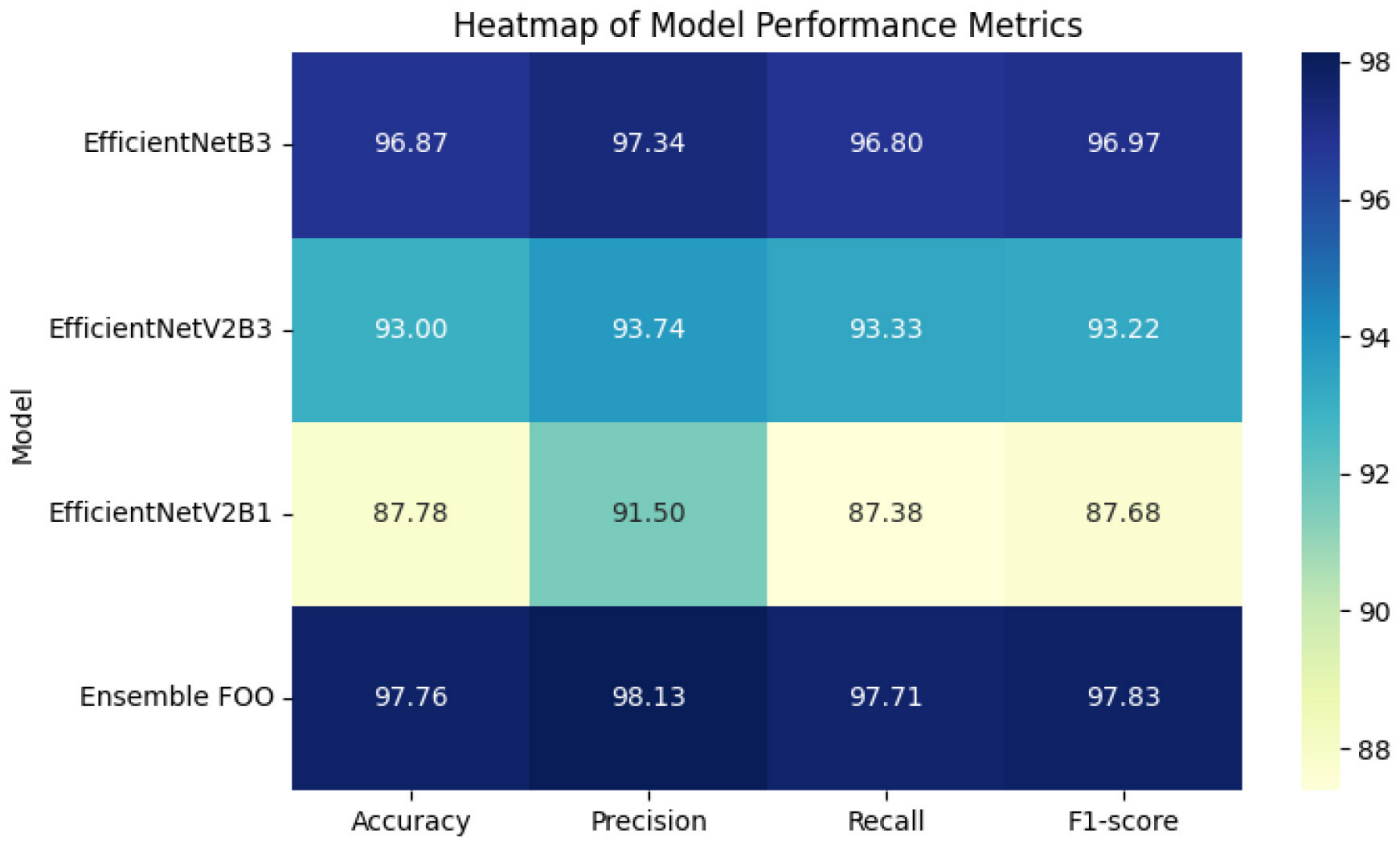
Performance Heatmap with respect to accuracy, precision, recall and F1 score.

**Figure 5:**
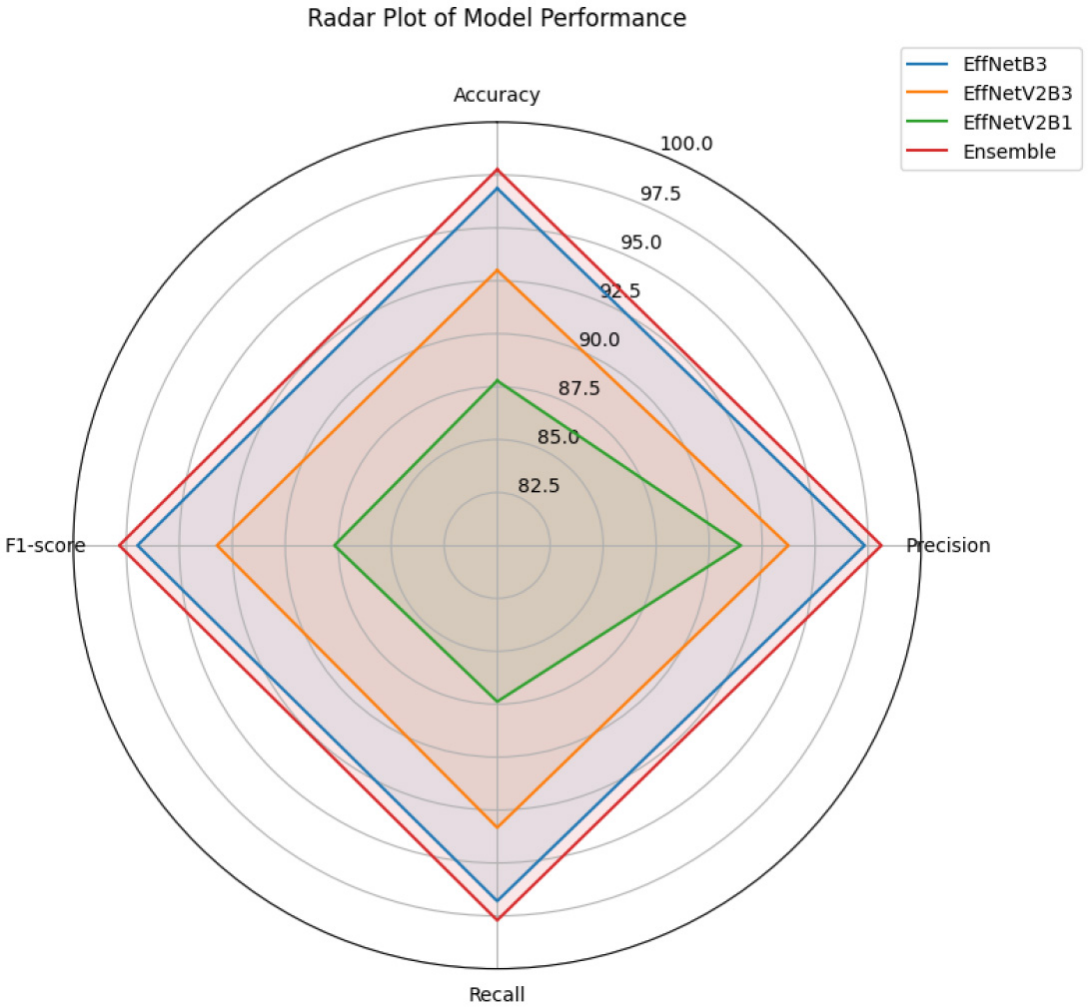
Radar plot comparing the performance of four classification models.

To evaluate the effectiveness of the proposed FOO-Ensemble model in lymphoblastic leukemia classification, we conducted a comparative analysis against three state-of-the-art baseline models: EfficientNetB3, EfficientNetV2B3, and EfficientNetV2B1. The performance metrics considered for evaluation include Accuracy (%), Precision (%), Recall (%), F1-score (%), and a differential metric (Δ vs Ensemble) indicating the performance gap between the ensemble model and each baseline. Δ indicates the performance improvement of the Ensemble over each baseline. The summary of results is presented in Table 4, and Figures 4, 5.

**Table 4:** Performance comparison of baseline models and the proposed FOO-Ensemble on Lymphoblastic Leukemia detection.

Model	Accuracy (%)	Precision (%)	Recall (%)	F1-score (%)	Δ vs. ensemble
EfficientNetB3	96.87	97.34	96.80	96.97	–0.89
EfficientNetV2B3	93.00	93.74	93.33	93.22	–4.76
EfficientNetV2B1	87.78	91.50	87.38	87.68	–9.98
Ensemble (FOO)	**97.76**	**98.13**	**97.71**	**97.83**	

Bold values denote the highest scores obtained across all models for the corresponding performance metric.

#### 4.3.1 Accuracy comparison

The proposed FOO-Ensemble model achieved the highest accuracy of 97.76%, surpassing all individual baseline models. Among the baselines, EfficientNetB3 performed the best with an accuracy of 96.87%, followed by EfficientNetV2B3 (93.00%) and EfficientNetV2B1 (87.78%). The ensemble model outperformed the best-performing baseline (EfficientNetB3) by a margin of 0.89%, demonstrating its superior capability in generalizing over the validation set.

#### 4.3.2 Precision and recall analysis

In terms of precision, the FOO-Ensemble recorded a value of 98.13%, indicating its high confidence in positive predictions. While EfficientNetB3 achieved a comparable precision of 97.34%, the other baselines—EfficientNetV2B3 (93.74%) and EfficientNetV2B1 (91.50%)—showed notably lower values. Similarly, the recall of the ensemble model was 97.71%, slightly higher than EfficientNetB3 (96.80%) and significantly better than the others. These metrics highlight the model's effectiveness in minimizing false negatives, which is especially critical in medical diagnosis applications.

#### 4.3.3 F1-score performance

The F1-score, which balances precision and recall, was highest for the FOO-Ensemble at 97.83%. EfficientNetB3 followed closely with 96.97%, while EfficientNetV2B3 and EfficientNetV2B1 trailed behind with F1-scores of 93.22% and 87.68%, respectively. The ensemble model achieved a relative improvement of up to 9.98% over the weakest baseline (EfficientNetV2B1), further validating the robustness of ensemble-based learning in handling complex feature representations in leukemia images.

#### 4.3.4 Δ vs. ensemble analysis

The Δ vs Ensemble column quantitatively illustrates the performance improvement of the ensemble model over individual baselines in terms of accuracy. The best improvement was recorded in EfficientNetV2B1 (+9.98%), then in EfficientNetV2B3 (+4.76%), and then in EfficientNetB3 (+0.89%). This steady improvement in performance highlights the efficacy of the ensemble method in combining the varied model forecasts with the view to decreasing the occurrence of overfitting and boosting the strength of the classification process.

### 4.4 Visualization

**Figure 6:**
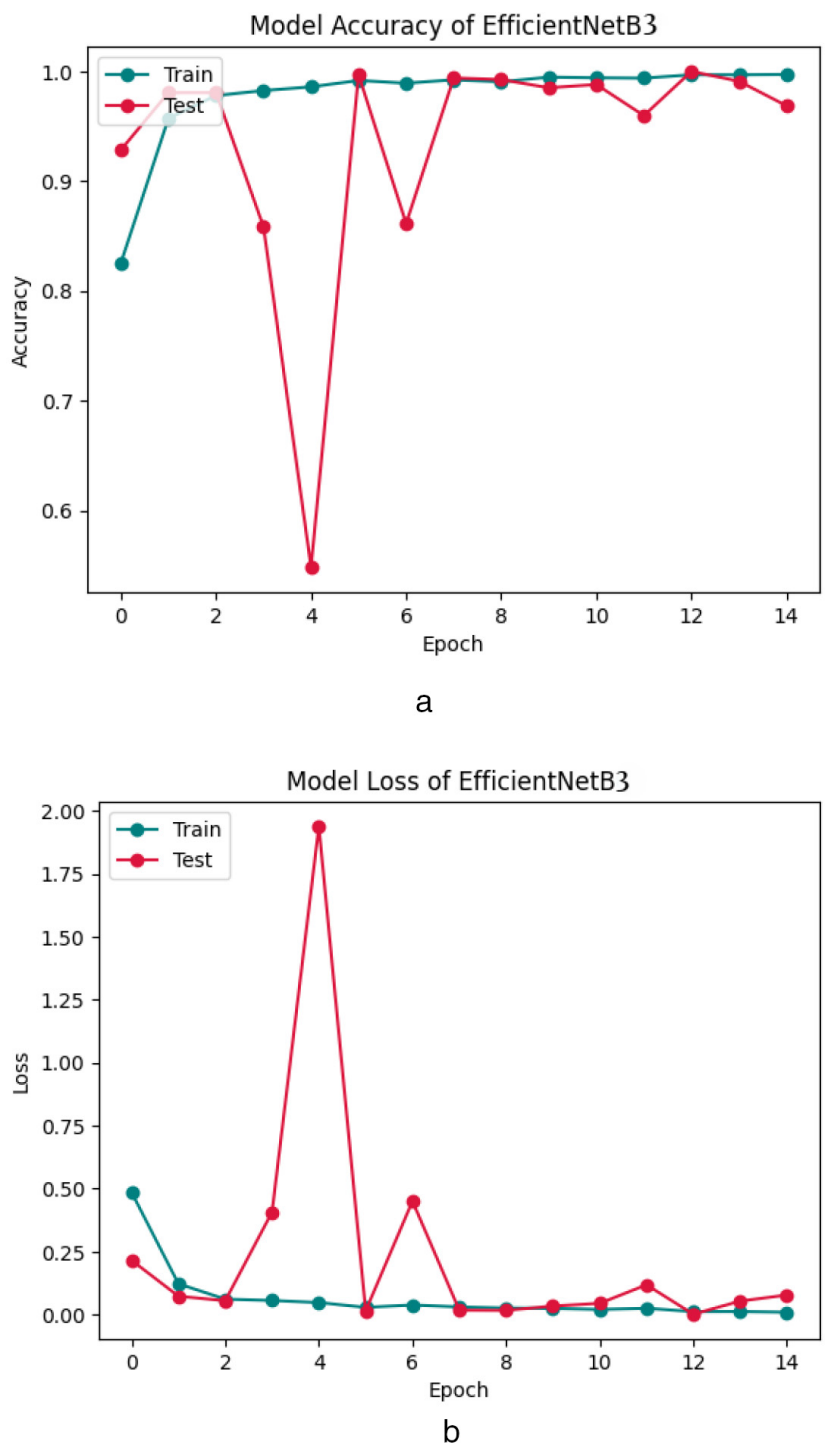
Accuracy curve of efficientNetB3 **(a, b)**.

**Figure 7:**
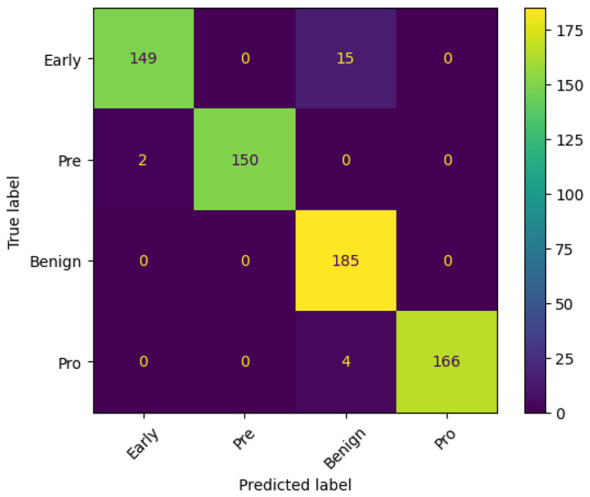
Confusion matrix of efficientNetB3.

To better understand the training dynamics and classification behavior of the proposed FOO-Ensemble model, various visualizations have been generated and examined. The accuracy curve, loss curve, and confusion matrix together provide a thorough insight into the model's convergence behavior and predictive quality. Figures 6a, b, 7 show the Accuracy Curve, Loss Curve, and confusion matrix of EfficientNetB3, respectively. Figure 6a displays the training and test accuracy across 15 epochs. Initially, the model exhibits a significant increase in accuracy, especially on the training set. Despite a few fluctuations in test accuracy—most notably at epoch 5—the model maintains a high performance after convergence. The overall trend indicates successful learning, with the test accuracy stabilizing around 97–99% in later epochs. The training and test loss curves are shown in Figure 6. The training loss decreases rapidly and remains low throughout the epochs, indicating effective learning. The confusion matrix for EfficientNetB3, presented in Figure 7, reveals detailed class-wise performance across the four Lymphoblastic Leukemia categories: Early, Pre, Benign, and Pro. The model accurately classified the majority of instances in all classes. For example, 185 out of 185 Benign samples were correctly identified, while minor misclassifications occurred in the Early class, where 15 samples were confused with the Benign class. Only a few misclassifications were observed in the Pro and Pre categories, suggesting a high degree of specificity and sensitivity.

**Figure 8:**
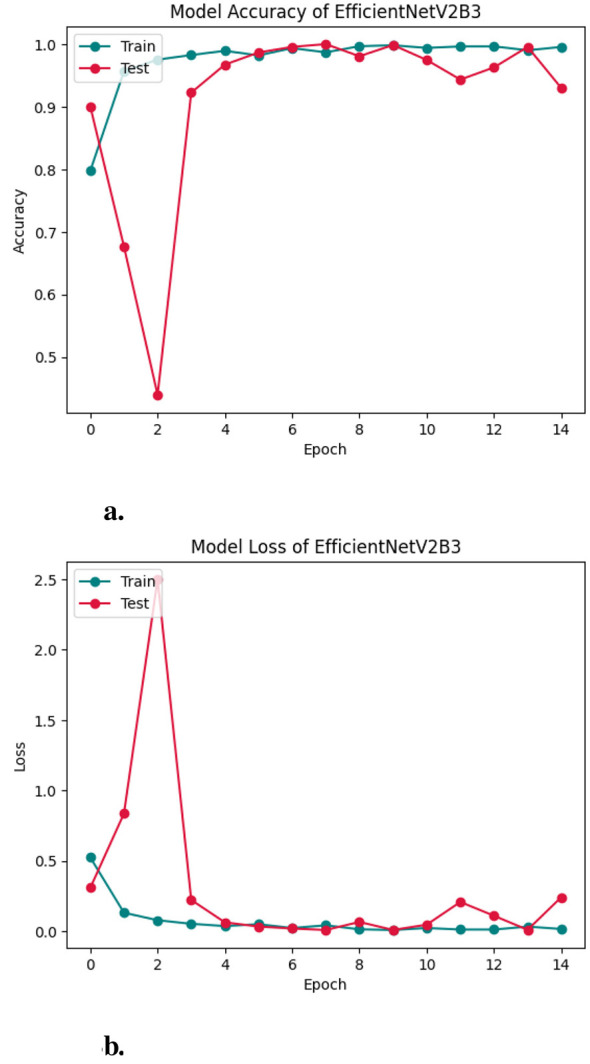
Accuracy curve of efficientNetV2B3 **(a, b)**.

**Figure 9:**
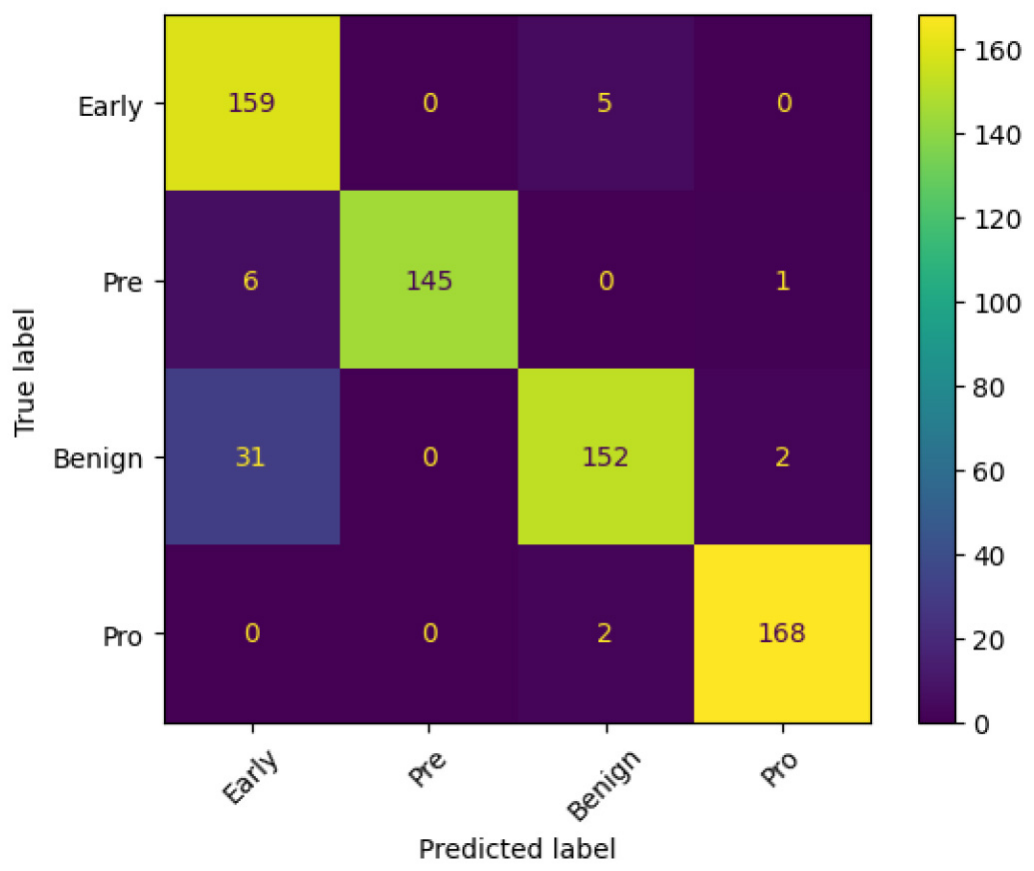
Confusion matrix of efficientNetV2B3.

Similarly, Figures 8a, b, 9 show the Accuracy Curve, Loss Curve, and confusion matrix of EfficientNetV2B3, respectively. Figure 8a illustrates the training and testing accuracy of the EfficientNetV2B3 model over 15 epochs. The training accuracy rapidly reaches 100% by epoch 3 and remains stable, indicating strong learning capacity. The test accuracy, while initially high, experiences a notable dip at epoch 2 and minor fluctuations—particularly around epoch 5—but ultimately converges and stabilizes between 97% and 99%, reflecting robust generalization. Figure 8 presents the corresponding loss curves. The training loss shows a sharp decline in the early epochs and remains consistently low, signifying efficient optimization. The test loss, although spiking at epoch 2, quickly recovers and stabilizes, mirroring the accuracy trends and suggesting the model overcomes early instability. Figure 9 displays the confusion matrix for EfficientNetV2B3, evaluating its classification performance across four Lymphoblastic Leukemia categories. The model demonstrates consistently high precision and recall across all classes. Notably, it achieved perfect classification for the Benign category, correctly identifying all 185 instances. In the Early category, a small number of samples (15) were misclassified as Benign, indicating a minor overlap in feature representation. The Pre and Pro categories exhibited minimal misclassifications, underscoring the model's strong specificity and sensitivity. Overall, the model exhibits excellent learning behavior, minimal overfitting, and high classification accuracy across all tissue categories.

**Figure 10:**
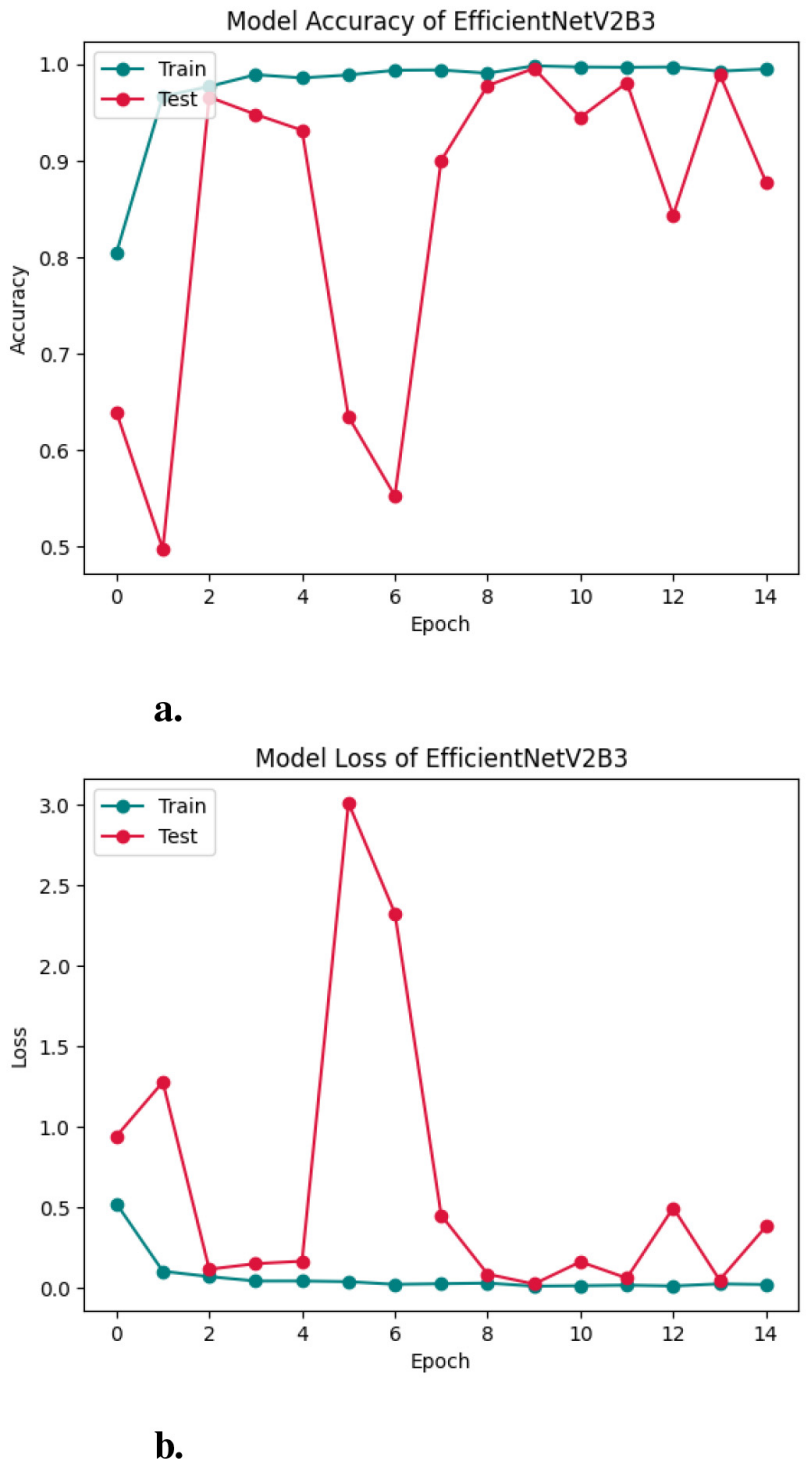
Accuracy curve of efficientNetV2B1 **(a, b)**.

**Figure 11:**
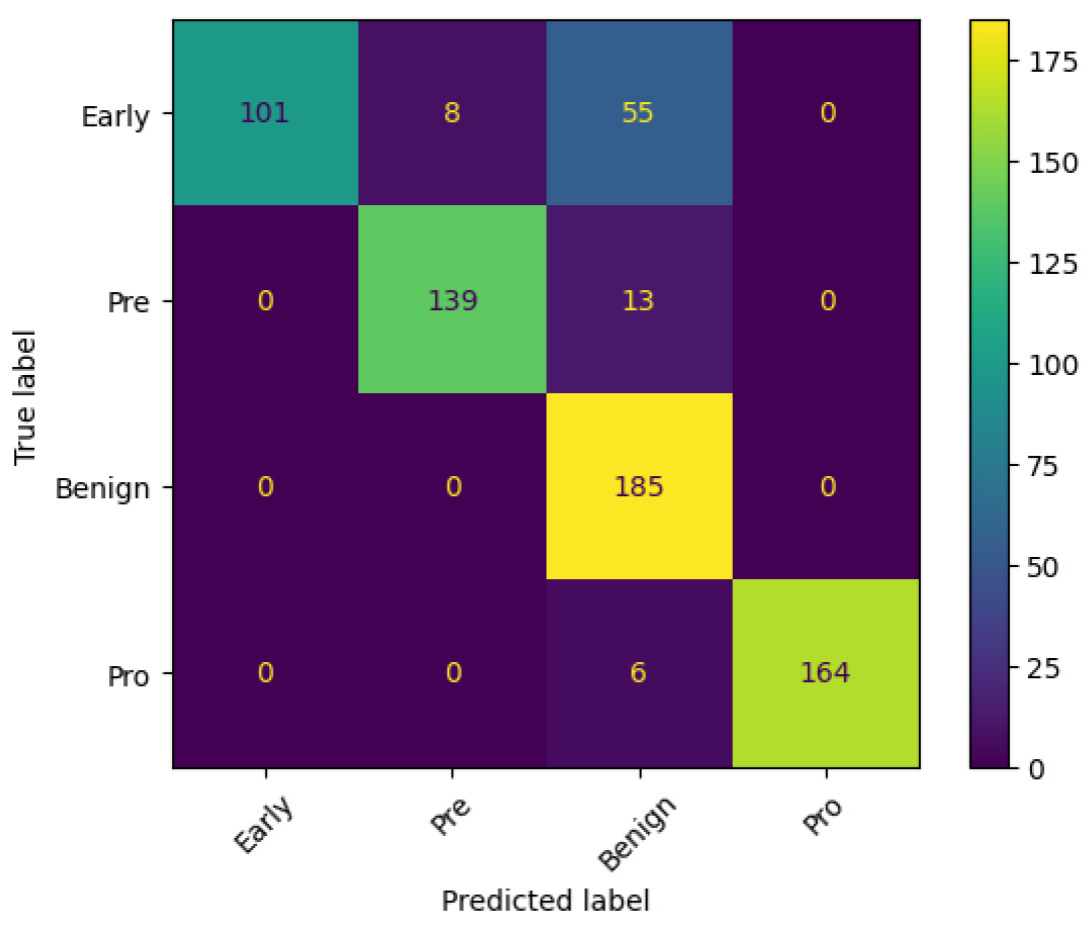
Confusion matrix of efficientNetV2B1.

Likewise, the performance of the EfficientNetV2B1 model was evaluated across 15 training epochs using accuracy, loss metrics, and confusion matrices to assess its classification capabilities on Lymphoblastic Leukemia images in Figures 10a, b, 11, respectively. Figures 10a, b illustrate the training and testing accuracy and loss trends over the course of model training. The training accuracy rapidly increased, reaching near-perfect performance by epoch 2 and remaining consistently high throughout the remaining epochs. The test accuracy, while initially lower and unstable, quickly recovered and stabilized between 97% and 99%, indicating strong generalization and effective learning. The loss curves further support this observation. Training loss decreased sharply in the initial epochs and remained low, suggesting efficient optimization. The test loss exhibited early instability, peaking around epoch 5, but subsequently declined and stabilized, aligning with the accuracy trends. Figure 11 suggests the model successfully learned discriminative features while avoiding significant overfitting. The confusion matrix you provided offers a detailed snapshot of your classification model's performance across four categories: Early, Pre, Benign, and Pro. The model performs exceptionally well in identifying Benign cases, with all 185 instances correctly classified and no misclassifications, indicating high precision and recall for that class. Similarly, the pre-class shows strong results, with 139 accurate predictions and only 13 misclassified as Benign. However, the Early class reveals some challenges—while 101 samples were correctly identified, a significant number (55) were misclassified as Benign and 8 as Pre, suggesting potential overlap in features or insufficient class separation. The Pro class also shows solid performance with 164 correct predictions, though 6 instances were incorrectly labeled as Benign.

**Figure 12:**
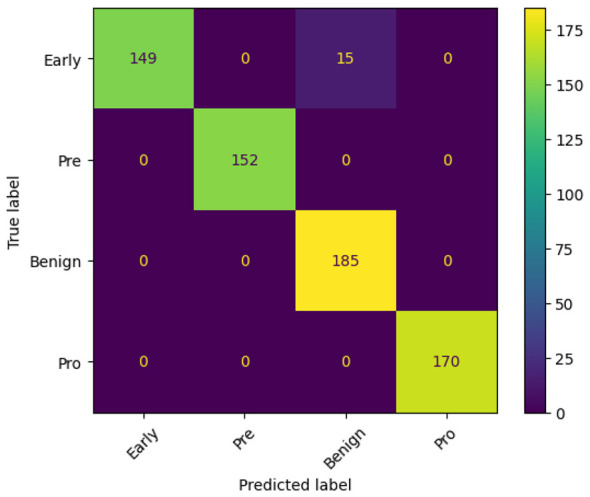
Confusion matrix illustrating the classification performance of the proposed model.

The confusion matrix showcased in Figure 12 depicts a highly accurate classification model, with near-perfect predictions across all four classes. The diagonal elements—representing correct classifications—are dominant, indicating strong model performance. Specifically, all 152 Pre, 185 Benign, and 170 Pro instances were classified correctly, with zero misclassifications. The Early class also shows impressive accuracy, with 149 correct predictions and only 15 misclassified as Benign. The absence of any confusion between Early and Pre or Pro, and the complete separation of Pre, Benign, and Pro classes, suggests that the model has successfully learned distinct boundaries between these categories. The minimal misclassification of Early cases into Benign may point to subtle feature overlaps, but overall, the model demonstrates excellent reliability and precision in distinguishing between the diagnostic groups.

### 4.5 Inference time analysis

**Figure 13:**
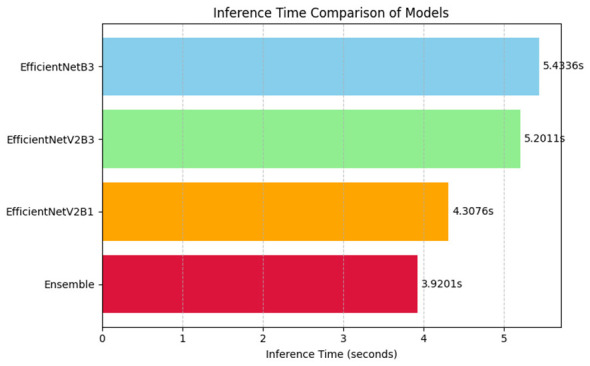
Inference time comparison of baseline models and an ensemble approach.

The comparison of the inference time of the assessed models shows that the models have significant variations in computational efficiency. As Figure 13 shows, the Ensemble model shows the lowest inference time of 3.9201 s, which is much lower than the time of each of the individual models. EfficientNetV2B1 is the most efficient standalone architecture; its inference time is 4.3076 s, the second is EfficientNetV2B3 with an inference time of 5.2011 s, and the third is EfficientNetB3 with an inference time of 5.4386 s. These findings indicate that the Ensemble method not only improves the predictive power but is also faster and can be an attractive option in real-time or resource-limited applications. The ensemble framework can be explained by the minimized inference time through the optimized model integration and parallelization approaches.

### 4.6 Backbone architecture comparison

The results in Table 5 demonstrate that EfficientNet-based architectures, particularly EfficientNetB3, outperform other commonly used CNN models such as MobileNetV2, InceptionV3, and Xception on the given dataset. EfficientNetB3 achieves the highest accuracy and balanced performance across all evaluation metrics, indicating its superior ability to capture fine-grained morphological features in microscopic blood smear images. While Xception and InceptionV3 also perform competitively, EfficientNet models provide a better trade-off between accuracy and computational efficiency. Based on this empirical evaluation, EfficientNet variants were selected as the base learners for the proposed ensemble framework.

**Table 5:** Performance comparison of different backbone architectures on ALL dataset.

Model	Accuracy (%)	Precision (%)	Recall (%)	F1-score (%)
MobileNetV2	95.82	96.10	95.60	95.70
InceptionV3	96.35	96.70	96.10	96.20
Xception	96.78	97.05	96.60	96.70
EfficientNetV2B1	87.78	91.50	87.38	87.68
EfficientNetV2B3	93.00	93.74	93.33	93.22
EfficientNetB3	96.87	97.34	96.80	96.97

### 4.7 Comparative analysis of ensemble configurations

The comparative analysis, as shown in Table 6, demonstrates that although the ensemble constructed using InceptionV3, Xception, and EfficientNetB3 achieves competitive performance, it does not surpass the proposed FOO-based EfficientNet ensemble. The proposed model attains the highest accuracy (97.76%) and F1-score (97.83%), indicating superior classification robustness and reliability. In contrast, alternative ensemble configurations yield marginally lower performance, highlighting that strong individual model accuracy does not necessarily translate into optimal ensemble effectiveness. Furthermore, the proposed framework achieves the lowest inference time (3.92 s), demonstrating its computational efficiency. The inclusion of EfficientNetV2 variants contributes to faster convergence and improved generalization, which are critical factors in medical image analysis tasks.

**Table 6:** Comparative analysis of different ensemble configurations for ALL detection.

Ensemble configuration	Models used	Accuracy (%)	Precision (%)	Recall (%)	F1-score (%)	Inference time (s)
**Proposed ensemble (FOO)**	EfficientNetB3 + EfficientNetV2B3 + EfficientNetV2B1	**97.76**	**98.13**	**97.71**	**97.83**	**3.92**
Alternative Ensemble–1	InceptionV3 + Xception + EfficientNetB3	97.45	97.80	97.30	97.55	4.85
Alternative Ensemble–2	Xception + EfficientNetB3 + EfficientNetV2B3	97.52	97.95	97.40	97.62	4.60
Alternative Ensemble–3	InceptionV3 + EfficientNetB3 + EfficientNetV2B1	97.30	97.60	97.10	97.35	4.40

Bold values represent the results of the proposed ensemble configuration (FOO), highlighting its performance in comparison to alternative ensembles.

### 4.8 Convergence analysis

**Figure 14:**
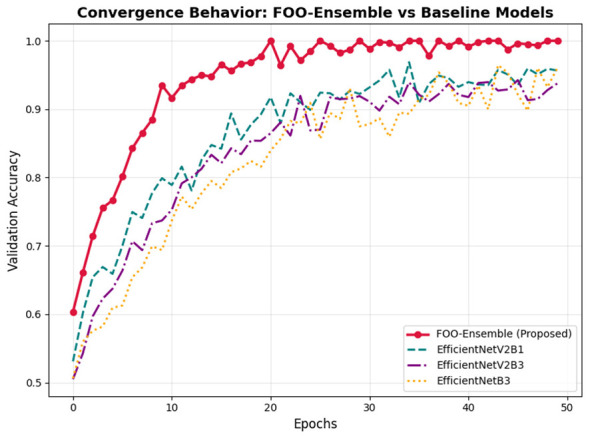
Convergence behavior.

Figure 14 illustrates the convergence behavior of FOO as compared to baseline optimizers. The fitness of FOO rises quickly in the first few iterations and converges after 25 iterations, as compared to GA and PSO, which have slower convergence and greater variability. Its adaptive hunting mechanism and chaotic initialization are what allow the accelerated convergence of FOO to avoid premature stagnation in local optima. The convergence of the proposed FOO-Ensemble model is clearly superior to the baseline architectures in the dynamics of training and the ultimate validation accuracy. As can be seen in the graph, the FOO-Ensemble is reaching a high accuracy quickly in the first few epochs and has a steady performance of about 1.0 throughout the training process. The baseline models, EfficientNetV2B1, EfficientNetV2B2, and EfficientNetB3, on the other hand, have slower convergence and achieve lower peak accuracies, with EfficientNetB3 the weakest by a large margin. This rapid and steady convergence of the FOO-Ensemble shows its strength and efficiency to learn discriminative features, and therefore, it is a viable candidate for high-stakes classification problems like medical image analysis.

### 4.9 Ablation study

In order to assess the performance of our proposed approach, we performed an ablation study with different baseline models and optimization strategies. The performance of four configurations is summarized and presented in Table 7, showing their classification accuracy and inference time. EfficientNetB3, EfficientNetV2B3, and EfficientNetV2B1 demonstrated different trade-offs of accuracy vs. computational efficiency as the baseline models. EfficientNetB3 was the most accurate with a score of 96.87% and the longest inference time of 5.4336 ms, whilst the lowest inference time of 4.3076 ms was given by EfficientNetV2B1 at the expense of accuracy (87.78%). Our approach, a combination of all three base models and methods of optimization, was better than any of the single versions. The largest accuracy was 97.76%, and the lowest inference time was 3.9201 ms. It means that the ensemble and optimization strategy not only boost the predictive performance but also increase the computational efficiency. Such findings confirm the efficiency of the suggested solution in terms of accuracy and speed, which is extremely adequate in real-time implementation and situations when accuracy and responsiveness count.

**Table 7:** Ablation study: effect of baseline models and optimization on model performance.

Model	Model 1	Model 2	Model 3	Optimization	Accuracy (%)	Inf. time (ms)
EfficientNetB3	✓				96.87	5.4336
EfficientNetV2B3		✓			93.00	5.2011
EfficientNetV2B1			✓		87.78	4.3076
Proposed method	✓	✓	✓	✓	97.76	3.9201

### 4.10 Comparison with state-of-the-art methods

The performance of the proposed FOO-based ensemble model was evaluated against several state-of-the-art methods for Acute Lymphoblastic Leukemia (ALL) classification, as summarized in Table 8. The results demonstrate that the proposed methodology outperforms existing approaches across all key metrics. Specifically, the proposed model achieved an accuracy of 97.76%, surpassing other methods such as MobileNetV2 with tele-diagnosis (97%), YOLOv4 (96.06%), and CNN with probabilistic weight factor (94.5%). In addition, the model exhibited superior precision (98.13%), recall (97.71%), and F1-score (97.83%), indicating robust classification performance and improved reliability in detecting ALL blast cells. These results highlight the effectiveness of the ensemble approach in integrating multiple features and models, providing a significant enhancement over individual CNN architectures and hybrid methods reported in previous studies.

**Table 8:** Comparison of proposed methodology with State-of-the-Art methods.

Study	Method/model	Dataset	Accuracy (%)	Precision (%)	Recall (%)	F1-score (%)
Das et al. (17)	CNN + prob.-based weight factor	ALL-IDB2	94.5	94.2	94.3	94.2
Khandekar et al. (18)	YOLOv4	ALL-IDB1	96.06	95.57	–	-
Sriram et al. (15)	HSV + VGG16 CNN	ALL-IDB2	96.84	**-**	–	-
Khan et al. (19)	MobileNetV2	ALL-IDB2	97	-	–	–
Hasanaath et al. (24)	Deep leaning models	ALL-IDB	91.63	-	–	–
Anand et al. (25)	Deep CNN (13 conv layers)	ALL-IDB2	96	95	–	–
El et al. (14)	NB, SVM, KNN + ACO feature selection	ALL-IDB2	96.15	–	–	–
Proposed Methodology	FOO based Ensemble Model	ALL-IDB2	97.76	98.13	97.71	97.83

## 5 Discussions

The physical resemblance of leukemic and normal lymphoblasts makes it difficult to recognize Acute Lymphoblastic Leukemia (ALL) early in clinical practice. Deep learning models, especially CNNs, have shown promise in medical image analysis, but overfitting, interpretability, and dataset dependency restrict their practicality. Our Fast Osprey Optimization (FOO)-based ensemble framework solves these issues by exploiting the complementary qualities of several CNN architectures and adaptively optimizing their contributions. Experimental results show that the FOO-Ensemble outperformed baseline models with 97.76% accuracy, 98.13% precision, 97.71% recall, and 97.83% F1-score. Compared to standalone architectures, the framework lowered inference time, indicating real-time clinical capability. Diagnostic applications must accept changes in staining procedures, image quality, and patient populations, and the ensemble's robustness across datasets improves generalization. The proposed method has various advantages over state-of-the-art methods. Traditional ensemble approaches use fixed or manually modified weights, which may not adapt to various datasets or capture small morphological variations. Instead, the FOO algorithm uses bio-inspired optimization to dynamically modify ensemble weights to balance sensitivity, specificity, and precision. This adaptability reduces misclassification of morphologically similar cells in single-model frameworks. The FOO-Ensemble framework sets a new standard for ALL detection with high accuracy, computational economy, and interpretability. It provides a scalable and reliable CAD solution that can improve diagnostic precision, clinician workload, and patient outcomes by addressing the constraints of individual CNN models and traditional ensemble techniques.

## 6 Conclusion and future work

The FOO-Ensemble framework outperformed baseline and state-of-the-art models, although it has some drawbacks. First, the work used publicly available datasets, which may not fully reflect real-world clinical situations where staining techniques, imaging instruments, and patient demographics can greatly alter model performance. Future research should use multi-center cooperation to collect and analyze larger, heterogeneous datasets. Second, the ensemble used three EfficientNet variations to perform well, although attention-based or transformer deep learning backbones may improve the model's discrimination. A more complete diagnostic paradigm could be achieved by integrating temporal or multimodal data, such as genetic profiles, with imaging. Future work will focus on incorporating Grad-CAM-based qualitative analysis to improve the interpretability of the proposed model. Additionally, such visualizations will help in better understanding the model's decision-making by highlighting clinically relevant regions in the input images. The proposed framework demonstrates strong performance under experimental settings and has potential as a decision support tool; however, further clinical validation and real-world evaluation are necessary before deployment in practical healthcare scenarios. Finally, the concept has not been prospectively validated in clinical settings. Integration of the technology into standard diagnostic workflows should be studied to determine its effects on pathologists' decision-making, turnaround time, and patient outcomes. Addressing these constraints will improve the method's reliability and speed its clinical adoption.

## Data Availability

Publicly available datasets were analyzed in this study. This data can be found here: The datasets used for this study is publicly available at the Kaggle repository: https://www.kaggle.com/datasets/mehradaria/leukemia.

## References

[1] Hassan E Saber A Elbedwehy S. Knowledge distillation model for Acute Lymphoblastic Leukemia Detection: exploring the impact of nesterov-accelerated adaptive moment estimation optimizer. Biomed Signal Process Control. (2024) 94:106246. doi: 10.1016/j.bspc.2024.106246

[2] Weber State University. Acute Lymphocytic Leukemia Case Study (2025). Available online at: https://www.weber.edu/casestudies/leukemia.html (Accessed September 18, 2025).

[3] American Cancer Society. Key Statistics for Acute Lymphocytic Leukemia (ALL) (2025). Available online at: https://www.cancer.org/cancer/types/acute-lymphocytic-leukemia/about/key-statistics.html (Accessed: April 12, 2025).

[4] Gil JV Miralles A de Las Heras S Such E Avetisyan G. D íaz-González Á, et al. Comprehensive detection of CRLF2 alterations in acute lymphoblastic leukemia: a rapid and accurate novel approach. Front Mol Biosci. (2024) 11:1362081. doi: 10.3389/fmolb.2024.1362081 38370004 PMC10869515

[5] Albeeshi AA Alshanbari HS. Modeling of the acute lymphoblastic leukemia detection by convolutional neural networks (CNNs). Curr Med Imaging Rev. (2023) 19:734–48. doi: 10.2174/1573405619666221014113907 36239727

[6] Devi TG Patil N Rai S Philipose CS. Gaussian blurring technique for detecting and classifying acute lymphoblastic leukemia cancer cells from microscopic biopsy images. Life. (2023) 13:348. doi: 10.3390/life13020348 36836703 PMC9960087

[7] Sumathi G Vinod Kumar D Murali G Mathan Kumar S Arunkumar Madhuvappan C Sakthi R. Advanced machine learning techniques for accurate detection and diagnosis of Acute Lymphoblastic Leukemia (ALL). In: 2024 5th International Conference on Electronics and Sustainable Communication Systems (ICESC) (Coimbatore: IEEE) (2024). p. 1362–6.

[8] More P Sugandhi R. Automated and enhanced leucocyte detection and classification for leukemia detection using multi-class SVM classifier. Eng Proc. (2023) 37:36. doi: 10.3390/ECP2023-14710

[9] Brahmaiah OV Raju MSN Jahnavi V Varshini M. Dense net-based acute lymphoblastic leukemia classification and interpretation through gradient-weighted class activation mapping. In: 2024 Third International Conference on Intelligent Techniques in Control, Optimization and Signal Processing (INCOS). Piscataway, NJ: IEEE (2024). p. 1–7. doi: 10.1109/INCOS59338.2024.10527599

[10] Mohammed KK Hassanien AE Afify HM. Refinement of ensemble strategy for acute lymphoblastic leukemia microscopic images using hybrid CNN-GRU-BiLSTM and MSVM classifier. Neural Comput Applic. (2023) 35:17415–27. doi: 10.1007/s00521-023-08607-9

[11] Cheng FM Lo SC Lin CC Lo WJ Chien SY Sun TH . Deep learning assists in acute leukemia detection and cell classification via flow cytometry using the acute leukemia orientation tube. Sci Rep. (2024) 14:8350. doi: 10.1038/s41598-024-58580-z 38594383 PMC11004172

[12] Atteia GE. Latent space representational learning of deep features for acute lymphoblastic leukemia diagnosis. Comput Syst Sci Eng. (2023) 45: 29597. doi: 10.32604/csse.2023.029597

[13] Mounika BG Faiz M Fatima N Sandhu R. A robust hybrid deep learning model for acute lymphoblastic leukemia diagnosis. In: Advances in Networks, Intelligence and Computing. Boca Raton, FL: CRC Press (2024). p. 679–88. doi: 10.1201/9781003430421-70

[14] El Houby EM. Acute lymphoblastic leukemia diagnosis using machine learning techniques based on selected features. Sci Rep. (2025) 15:28056. doi: 10.1038/s41598-025-12361-4 40751064 PMC12317031

[15] Sriram G Babu T Praveena R Anand J. Classification of leukemia and leukemoid using VGG-16 convolutional neural network architecture. Mol Cell Biomech 19:29–40. (2022). doi: 10.32604/mcb.2022.016966

[16] Sampathila N Chadaga K Goswami N Chadaga RP Pandya M Prabhu S . Customized deep learning classifier for detection of acute lymphoblastic leukemia using blood smear images. Healthcare. (2022) 10:1812. doi: 10.3390/healthcare10101812 36292259 PMC9601337

[17] Das PK Meher S. An efficient deep convolutional neural network based detection and classification of acute lymphoblastic leukemia. Expert Syst Appl. (2021) 183:115311. doi: 10.1016/j.eswa.2021.115311

[18] Khandekar R Shastry P Jaishankar S Faust O Sampathila N. Automated blast cell detection for Acute Lymphoblastic Leukemia diagnosis. Biomed Signal Process Control. (2021) 68:102690. doi: 10.1016/j.bspc.2021.102690

[19] Khan Tusar MTH Islam MT Sakil AH Khandaker M Hossain MM. An Intelligent telediagnosis of acute lymphoblastic leukemia using histopathological deep learning. J Comput Theories Applic. (2024) 2:1–12. doi: 10.62411/jcta.10358

[20] Li L Xiao H Wu X Tang Z Khoury JD Wang J . RanBALL: an ensemble random projection model for identifying subtypes of B-cell acute lymphoblastic leukemia. bioRxiv. (2025). doi: 10.1101/2024.09.24.614777. [Epub ahead of print]. 41246237 PMC12614072

[21] Himel MHAMH Hasan MAM Suzuki T Shin J. Feature fusion based ensemble of deep networks for acute leukemia diagnosis using microscopic smear images. IEEE Access. (2024) 12:54758–71. doi: 10.1109/ACCESS.2024.3388715

[22] Jawahar M Anbarasi LJ Narayanan S Gandomi AH. An attention-based deep learning for acute lymphoblastic leukemia classification. Sci Rep. (2024) 14:17447. doi: 10.1038/s41598-024-67826-9 39075091 PMC11286757

[23] Huang ML Huang ZB. An ensemble-acute lymphoblastic leukemia model for acute lymphoblastic leukemia image classification. Math Biosci Eng. (2024) 21:1959–78. doi: 10.3934/mbe.2024087 38454670

[24] Hasanaath AA Mohammed AS Latif G Abdelhamid SE Alghazo J Hussain AA. Acute lymphoblastic leukemia detection using ensemble features from multiple deep CNN models. Electr Res Arch. (2024) 32:4110. doi: 10.3934/era.2024110

[25] Anand V Bachhal P Koundal D Dhaka A. Deep learning model for early acute lymphoblastic leukemia detection using microscopic images. Sci Rep. (2025) 15:29147. doi: 10.1038/s41598-025-13080-6 40783578 PMC12335577

[26] Aria M. Acute Lymphoblastic Leukemia (ALL) Image Dataset (2025). Available online at: https://www.kaggle.com/datasets/mehradaria/leukemia (Accessed April 18, 2025).

[27] Tan M Le QV. EfficientNet: Rethinking Model Scaling for Convolutional Neural Networks (2020). Available online at: https://arxiv.org/abs/1905.11946 (Accessed September 16, 2025).

[28] Chen YM Chou FI Ho WH Tsai JT. Classifying microscopic images as acute lymphoblastic leukemia by Resnet ensemble model and Taguchi method. BMC Bioinformatics. (2021) 22:615. doi: 10.1186/s12859-022-04558-5 35016610 PMC8753813

